# Perioperative Management of Left Atrial Myxoma Causing Mitral Valve Obstruction and Thrombocytopenia: A Case Report

**DOI:** 10.7759/cureus.79978

**Published:** 2025-03-03

**Authors:** Ganesh Jayadevappa, Babar Chaudhri

**Affiliations:** 1 Department of Anaesthesiology, Aster Hospitals, Dubai, ARE; 2 Department of Cardiac Surgery, Aster Hospitals, Dubai, ARE

**Keywords:** cardiac tumor, left atrial myxoma, mitral valve obstruction, multidisciplinary management, thrombocytopenia

## Abstract

Left atrial myxomas are the most common primary cardiac tumors, often presenting with symptoms related to obstruction, embolization, or systemic effects. Thrombocytopenia is an uncommon but significant complication associated with myxomas, complicating their management. In this case, a 26-year-old male presented with severe dyspnea, palpitations, and blurred vision. Clinical examination revealed a mid-diastolic crescendo murmur, hepatomegaly, and elevated jugular venous pressure. Investigations showed thrombocytopenia, which is very significant in the perioperative management of this case, requiring specific treatment and precautions to be taken. The other factors, such as elevated liver enzymes and significant mitral valve obstruction caused by a left atrial mass, were also noted. Echocardiography and CT imaging confirmed the diagnosis of left atrial myxoma causing mitral valve inflow obstruction and hepatic congestion secondary to right heart failure. A multidisciplinary team managed the patient, and emergency surgery was performed to excise the tumor and repair the mitral and tricuspid valves with intraoperative transesophageal echocardiographic assistance. Postoperative management included monitoring in the intensive care unit, platelet transfusion, and supportive care, leading to a steady recovery. The patient was discharged on day 10 with stable platelet levels and total left atrial mass removal, which improved cardiac function. Histopathology confirmed the diagnosis of cardiac myxoma. This case highlights the diverse clinical manifestations of left atrial myxomas and the need for prompt diagnosis and multidisciplinary management, especially when complicated by thrombocytopenia and hepatic congestion. Surgical excision remains the treatment of choice, with favorable outcomes following complete tumor removal and valve repair.

## Introduction

Cardiac myxomas are the most common primary cardiac tumors, comprising approximately 50% of cases. They predominantly occur in the left atrium (75%), typically attaching to the atrial septum via a pedicle [1]. Although benign, their ability to obstruct blood flow or cause embolic events makes them clinically significant. The prevalence is about one in 10,000 individuals, with most cases diagnosed in adults aged 30-60, showing a female predominance [2,3].

Symptoms depend on the tumor’s size, mobility, and location. Common presentations include dyspnea, syncope, and palpitations, especially when the tumor obstructs the mitral valve during diastole, reducing cardiac output [4]. Embolic events such as transient ischemic attacks or peripheral embolisms may also occur if tumor fragments detach. Some patients may experience non-specific symptoms like fever, weight loss, or arthralgia, potentially due to cytokine release [5].

Less common manifestations, such as thrombocytopenia, have been reported. This complication may arise from mechanical platelet destruction, immune mechanisms, or tumor-induced inflammatory responses [6]. Thrombocytopenia complicates management, increasing hemorrhage risk during surgical intervention [7]. Understanding these varied presentations is crucial for timely diagnosis and management.

Echocardiography is the diagnostic gold standard, visualizing the mass’s size, mobility, and attachment site. Transthoracic echocardiography (TTE) is used initially, while transesophageal echocardiography (TEE) offers a more detailed view, especially for assessing valve involvement [8]. CT and MRI can further characterize the tumor and identify complications like embolism or myocardial invasion [9].

Histologically, myxomas appear gelatinous, composed of stellate cells within a mucopolysaccharide-rich stroma. Surgical resection is the preferred treatment, often performed urgently to prevent obstruction or embolism [10]. While recurrence is rare with complete excision, regular follow-up is necessary, particularly in familial cases linked to Carney complex [11].

This report discusses a young male with left atrial myxoma causing severe mitral obstruction and thrombocytopenia, leading to multi-system involvement, including hepatic congestion and right heart failure. A multidisciplinary approach was essential for optimizing outcomes. This case underscores the importance of early diagnosis and comprehensive management strategies, especially with atypical complications.

## Case presentation

Patient information

A 26-year-old male presented to the emergency department with complaints of severe breathlessness, which was aggravated on exertion and while lying flat. The patient reported episodes of palpitations, cough, and intermittent blurring of vision for the past few weeks. He had no significant past medical, surgical, or family history, and there was no history of smoking or alcohol consumption.

Clinical findings

On examination, the patient's vitals were as follows: BMI of 24.33 kg/m^2^, height of 160 cm, and weight of 62.3 kg. His pulse rate was 104 beats per minute, blood pressure was 102/67 mmHg, respiratory rate was 20 breaths per minute, and body temperature was 36.5°C. Oxygen saturation was recorded at 99% on room air. No pallor, edema, cyanosis, or petechiae were noted.

Cardiovascular examination revealed normal heart sounds (S1 and S2) with a mid-diastolic crescendo murmur and a raised jugular venous pulse. Respiratory examination showed bilateral basal crepitations, while neurological examination revealed the patient was conscious and oriented with no focal deficits. An abdominal examination indicated mild hepatomegaly with no evidence of free fluid or splenomegaly.

Diagnostic assessment

The patient was admitted to the intensive care unit (ICU) for further evaluation and management. Initial laboratory investigations revealed a low platelet count, elevated blood urea, elevated levels of serum glutamic-oxaloacetic transaminase (SGOT) and serum glutamic-pyruvic transaminase (SGPT), low albumin levels, and normal bilirubin levels (Table 1).

**Table 1:** Laboratory parameters SGOT: serum glutamic-oxaloacetic transaminase; SGPT: serum glutamic-pyruvic transaminase

Laboratory parameters	Values	Reference range
Haemoglobin	13.1 g/dL	13-17 g/dL
Platelet count	47,000/μL	150-450 x 10³/µL
Blood urea	62.43 mg/dL	19-44 mg/dL
Serum creatinine	0.78 mg/dL	0.6-1.2 mg/dL
SGOT	1902 U/L	8-33 U/L
SGPT	1285U/L	4-36 U/L
Albumin	5.3 g/dL	6.4-8.3 g/dL

**Figure 1:**
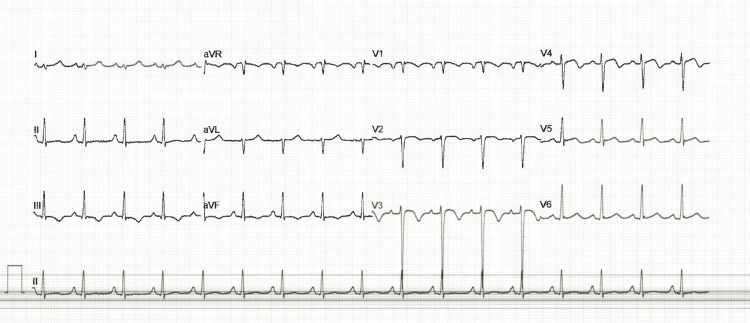
ECG findings of the patient

Electrocardiography (ECG) demonstrated sinus tachycardia, bi-atrial enlargement, right axis deviation, and T-wave inversion in leads III, aVF, and V2-V4, suggesting antero-inferior ischemia (Figure 1).

**Figure 2:**
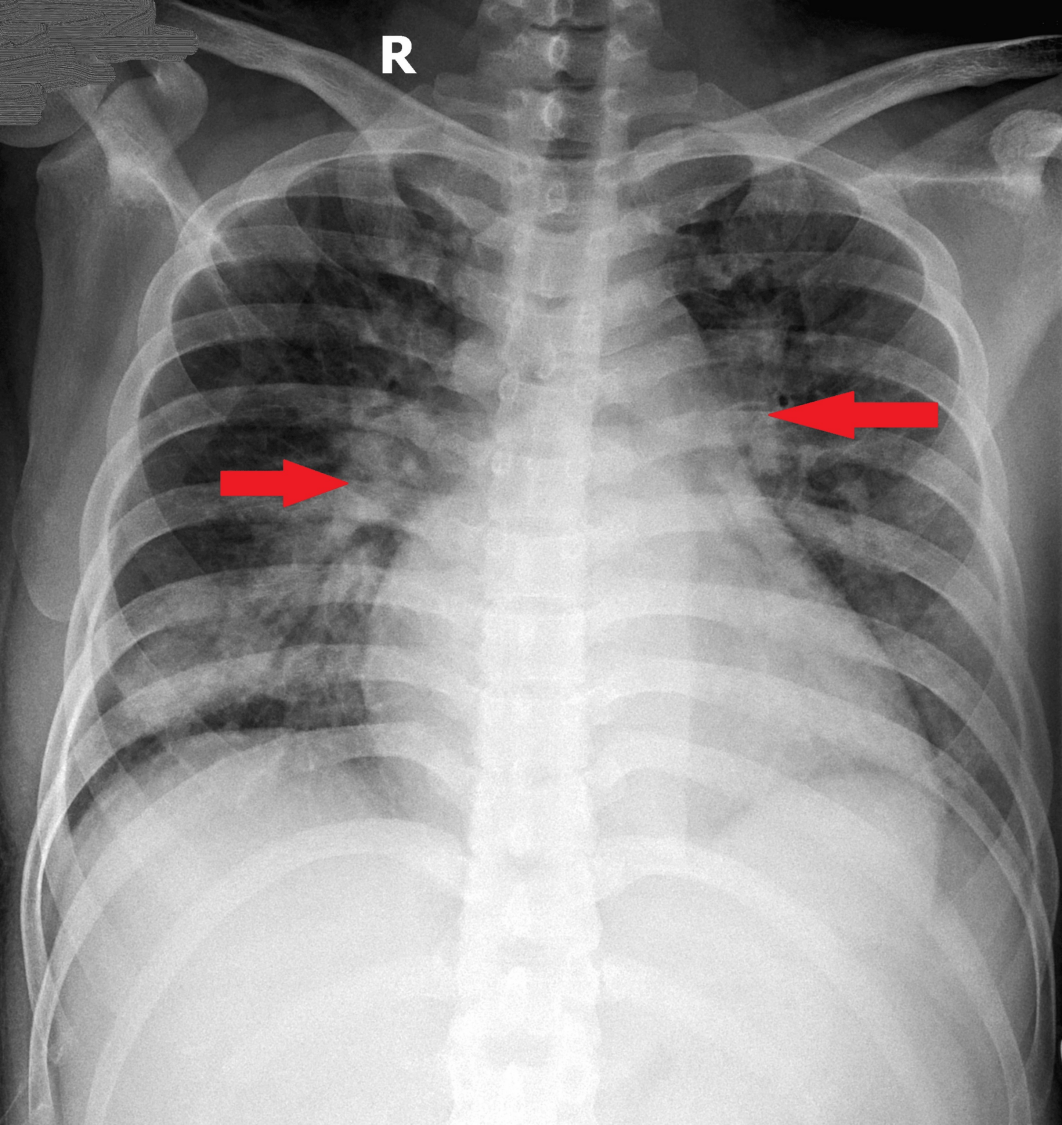
Chest X-ray showing bilateral perihilar consolidations (red arrows)

Chest X-ray showed bilateral perihilar consolidations (Figure 2, indicated by red arrows), suggesting an acute inflammatory or infectious process.

**Figure 3:**
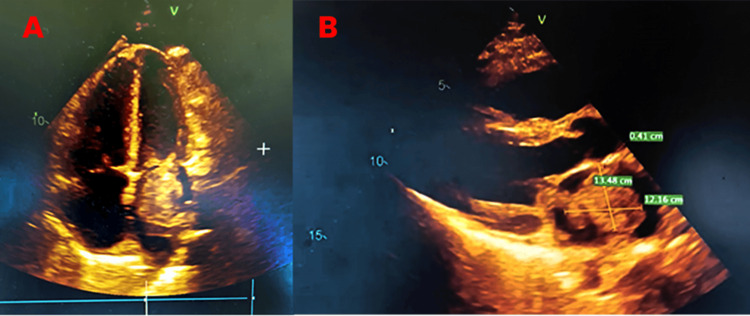
(A) Transthoracic echocardiography showing left atrial myxoma prolapsing into the mitral valve; (B) measurement of left atrial myxoma and mitral valve obstruction on echocardiography

TTE revealed a homogeneous, lobulated mass in the left atrium, prolapsing across the mitral valve and leading to moderate to severe mitral valve inflow obstruction, with a maximum pressure gradient (PG) of 28 mmHg and a mean PG of 10 mmHg (Figure 3).

Mild mitral regurgitation was also noted. The mass was attached to the posterior wall of the left atrium by a pedicle, suggestive of a left atrial myxoma. Left ventricular systolic function was normal, with a left ventricular ejection fraction (LVEF) of 62%, and no regional wall motion abnormalities were detected. Additionally, moderate pulmonary hypertension with mild tricuspid regurgitation was observed, with an estimated pulmonary artery systolic pressure (PASP) of 57 mmHg. Dilatation of the right atrium and right ventricle was also present, along with trace pericardial effusion.

**Figure 4:**
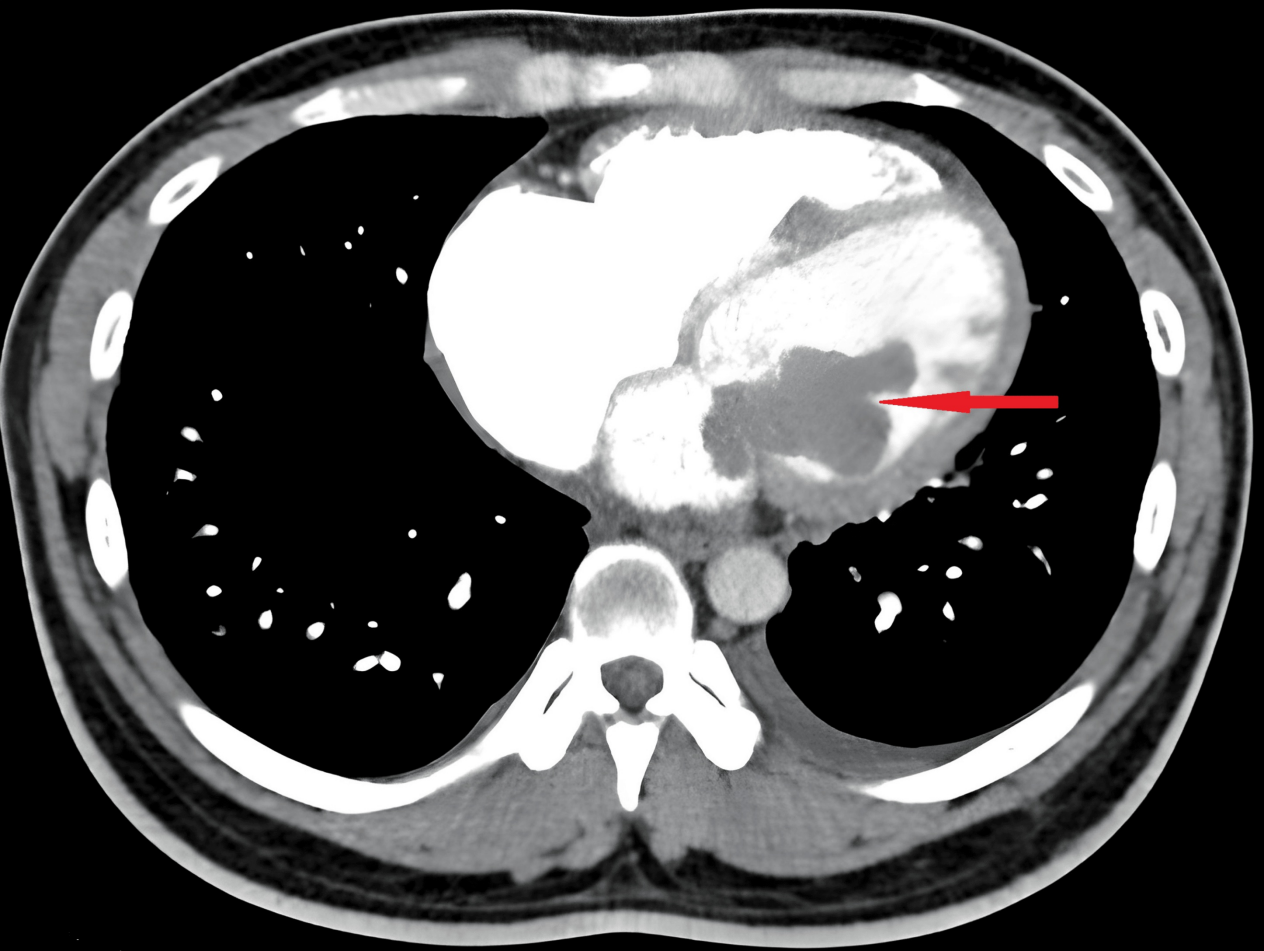
CT scan showing the presence of the left atrial mass prolapsing into the left ventricle (red arrow)

A computed tomography (CT) scan confirmed the presence of the left atrial mass prolapsing into the left ventricle, resulting in mild cardiomegaly and mild right lung basal interstitial edema (Figure 4, indicated by red arrow).

**Figure 5:**
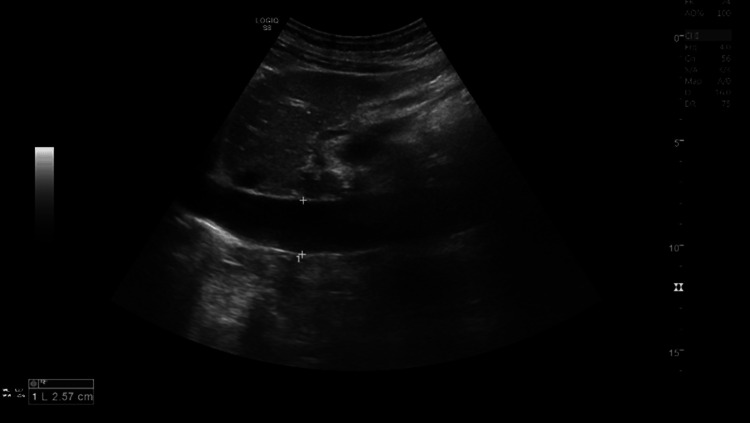
Abdominal ultrasound showing mild hepatomegaly

Hepatomegaly with heterogeneous venous phase enhancement, dilated inferior vena cava (IVC), and hepatic veins with reflux and delayed antegrade filling without thrombus were noted. There were features consistent with hepatic venous congestion secondary to right heart failure. An abdomen ultrasound showed mild hepatomegaly, increased periportal echoes, and pulsatile portal flow, all suggestive of cardiac congestion (Figure 5).

Mild bilateral pleural effusion was also identified. Despite platelet transfusions, the patient's platelet counts progressively dropped from 43,000 to 25,000/μL.

Final diagnosis

The diagnosis of a left atrial mass causing severe mitral valve obstruction, thrombocytopenia, hepatic congestion, and cardiac failure was confirmed. A multidisciplinary team, including a cardiothoracic surgeon, anesthesiologist, hematologist, gastroenterologist, and intensivist, was assembled to manage the patient. Given the critical condition, the team planned for urgent surgery to excise the left atrial mass and repair the mitral and tricuspid valves.

Therapeutic intervention

As recommended by the hematologist, the patient was started on hydrocortisone, dexamethasone, and romiplostim (a thrombopoietin receptor agonist) alongside platelet transfusions administered pre- and post-operatively. The gastroenterologist recommended maintaining cardiac function and reducing preload on the heart to alleviate hepatic congestion.

Preoperative preparation included two units of single donor platelet transfusion, which resulted in a temporary rise in platelet count. Under local anesthesia, ultrasound-guided central and arterial lines were established. The patient was monitored using ECG, SPO_2_, bispectral index (BIS), temperature, central venous pressure, and intra-arterial blood pressure. Anesthesia was induced using fentanyl (0.2 mcg/kg), midazolam (0.2 mg/kg), rocuronium (0.1 mg/kg), and morphine (0.1 mg/kg), following institutional protocols to maintain heart rate and blood pressure. An 8.0 mm cuffed endotracheal tube was used for intubation, and ventilation was initiated. The patient was maintained on sevoflurane (2%) and remifentanil infusion (0.02 mcg/kg/min), with intermittent boluses of rocuronium for muscle relaxation. TEE was used to reassess the mass’s location and origin, as well as to evaluate valvular obstruction and regurgitation. After coming off bypass, complete resection of the mass, valve repairs, and left ventricular function were reassessed.

The patient was fully heparinized with 400 units/kg of heparin, achieving an activated clotting time (ACT) above 480 seconds. Cardiopulmonary bypass was initiated using aortic and bicaval cannulae. The heart was arrested using Del Nido cardioplegia administered via the aortic root. The left atrium was opened, and the myxoma was successfully excised. The mitral valve was repaired using a semi-rigid ring, ensuring proper coaptation of the leaflets. The tricuspid valve was also repaired using a similar ring. After confirming adequate valve function, the heart was closed, and the patient was gradually weaned off cardiopulmonary bypass with the support of noradrenaline, levosimendan, and vasopressin. The mass excision and valve functions were confirmed through transoesophageal echocardiography.

The hepaire was fully reversed with protamine, and two units of single donor platelets were given to optimise the platelet count. The haemostasis was achieved, and the chest was closed.

Outcome and follow-up

Postoperatively, the patient was managed in the ICU with planned ventilation support. The patient’s condition stabilized, the bleeding was minimal, and he was extubated the following day. Laboratory parameters, including platelet count, showed steady improvement, and the patient was discharged on postoperative day 10 with a platelet count of 400,000/μL.

Histopathology

**Figure 6:**
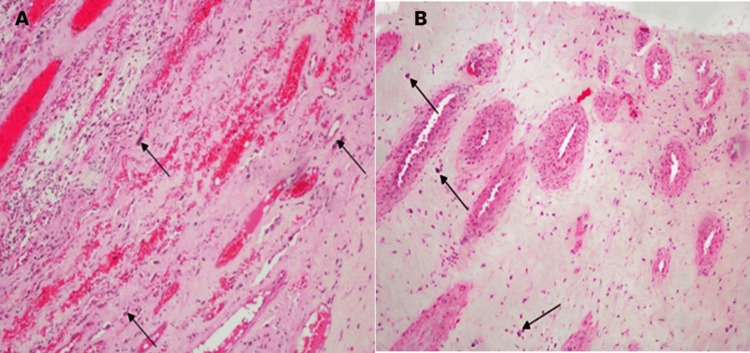
H&E stain of the histopathological section of the left atrial myxoma, showing polygonal and stellate cells in myxoid stroma at low magnification (A) and high magnification (B) (black arrows)

Histopathological analysis of the excised mass revealed a cauliflower-like, polypoidal structure measuring 5.5 x 4 x 2.5 cm and weighing 27 g. Sections showed a mass composed of polygonal and stellate cells embedded in a myxoid stroma containing chondroitin sulfate and hyaluronic acid, consistent with a diagnosis of cardiac myxoma (Figure 6, indicated by black arrows).

## Discussion

Left atrial myxoma is the most common primary cardiac tumor, with significant clinical implications due to its location and potential complications. Although myxomas are benign, their capacity to obstruct blood flow, cause embolic events, and manifest with systemic effects necessitates urgent diagnosis and intervention [2]. The case presented here illustrates the diverse clinical spectrum and complications associated with left atrial myxoma, particularly mitral valve obstruction and thrombocytopenia, which demanded a multidisciplinary approach for effective management.

Pathophysiology and clinical presentation

Left atrial myxomas are often attached to the interatrial septum, particularly at the fossa ovalis, and their size and mobility can cause dynamic obstruction of the mitral valve, leading to symptoms resembling those of mitral stenosis. The obstruction may worsen during diastole when the tumor prolapses into the mitral orifice, causing a reduction in cardiac output and leading to symptoms like dyspnea, orthopnea, and palpitations, as seen in this patient [4,6]. Other symptoms, such as cough and blurring of vision, as reported by the patient, may result from systemic embolization, which is a known complication of atrial myxomas. Tumor emboli can travel to the cerebral, coronary, or peripheral vasculature, leading to transient ischemic attacks, myocardial infarction, or limb ischemia [6].

Thrombocytopenia, observed in this case, is an atypical presentation of left atrial myxoma. It may occur due to several mechanisms, including immune-mediated platelet destruction, mechanical trauma caused by the tumor, or a systemic inflammatory response triggered by the tumor [7,8]. Studies have shown that in rare instances, cardiac tumors can alter hemodynamics and induce a hypercoagulable state, resulting in disseminated intravascular coagulation (DIC), which can further complicate the clinical scenario [8]. The falling platelet count despite transfusion, as seen in this patient, suggests an ongoing consumptive process, possibly exacerbated by the inflammatory cytokines released by the tumor.

Diagnostic modalities

The diagnosis of left atrial myxoma is often established through echocardiography, which is the most sensitive and specific diagnostic tool for detecting intracardiac masses. In this patient, TTE revealed a lobulated mass prolapsing into the mitral valve, leading to significant obstruction. This finding, along with the pressure gradients measured across the valve, was crucial in identifying the severity of the obstruction [9]. TEE could further enhance visualization, particularly in cases where TTE is inconclusive, by providing detailed information about the attachment, size, and mobility of the mass [10].

In addition to echocardiography, CT imaging is valuable for evaluating cardiac and extracardiac structures and identifying complications such as embolism or hepatic congestion, as seen in this case. The CT scan confirmed the presence of the mass and revealed hepatic venous congestion due to right heart failure, highlighting the systemic impact of the obstructive myxoma [11]. Such imaging modalities are essential not only for diagnosis but also for preoperative planning and assessing the extent of the disease.

Surgical management and outcomes

Surgical excision remains the definitive treatment for atrial myxomas, and the urgency of intervention depends on the presence of symptoms and hemodynamic compromise [2]. The surgical approach typically involves median sternotomy and cardiopulmonary bypass, as performed in this patient, allowing complete resection of the tumor and repair of any affected structures, such as the mitral valve [12]. In our patient, the mitral and tricuspid valves were repaired using semi-rigid rings to restore normal function and prevent further obstruction or regurgitation. Successful resection significantly reduces the risk of recurrence, which, although rare, has been reported in cases of incomplete excision or familial syndromes such as Carney complex [12,13].

The management of thrombocytopenia in the context of cardiac surgery posed an additional challenge. Perioperative bleeding is a major concern, particularly in patients with low platelet counts. In this case, the use of thrombopoietin analogs like romiplostim, combined with steroid therapy, helped stabilize the platelet count preoperatively [14]. This approach, along with intraoperative heparinization and careful monitoring using ACT, was crucial in minimizing the risk of hemorrhage. Multidisciplinary coordination among anaesthesiologists, cardiologists, hematologists, and surgeons was essential for optimizing the patient's condition perioperatively.

Postoperative management and histopathological findings

Postoperative care in such patients requires close monitoring in an ICU to manage potential complications like arrhythmias, bleeding, or valve dysfunction. In our patient, the planned postoperative ventilation and the gradual weaning off of inotropic support facilitated a stable recovery. The improvement in platelet count postoperatively indicates the resolution of the consumptive process, further supporting the hypothesis that the thrombocytopenia was tumor-related [8,14].

Histopathological examination remains the gold standard for confirming the diagnosis of myxoma. The characteristic appearance of stellate cells within a myxoid stroma and the presence of mucopolysaccharides such as hyaluronic acid and chondroitin sulfate were consistent with previous reports on the histology of atrial myxomas [15]. The absence of malignancy and clear margins following surgical excision is associated with a favorable prognosis, as seen in this case where the patient had an uneventful recovery and was discharged on the 10th postoperative day.

## Conclusions

This case emphasizes the importance of early detection and a comprehensive approach to managing left atrial myxomas. A rare clinical presentation like thrombocytopenia has to be evaluated and managed immediately for optimal outcome with the help of a multidisciplinary team to prevent perioperative bleeding. Though transthoracic echocardiography is the mainstay for the diagnosis of intracardiac masses, intraoperative assistance with transoesophageal echocardiography provides valuable information for precise surgical management. Timely surgical intervention with complete resection and repair of atrioventricular valves, combined with a coordinated multidisciplinary effort with intensive postoperative monitoring, is crucial for improving outcomes in such complex cases.
